# A 2-D Fully Polarized Van Atta Array Based on Wide-Beam Tri-Polarized Antennas

**DOI:** 10.3390/mi15111400

**Published:** 2024-11-20

**Authors:** Jicheng Pan, Lei Chen, Shuangdi Zhao, Tianling Zhang

**Affiliations:** 1 Guangzhou Institute of Technology, Xidian University, Xi’an 710071, China; jcpan0719@163.com; 2 National Key Laboratory of Radar Detection and Sensing, Xidian University, Xi’an 710071, China; zhaoshuangdi@stu.xidian.edu.cn (S.Z.); tianlingzhang@126.com (T.Z.)

**Keywords:** retrodirective array, wide-beam, tri-polarized antenna, 2-D Van Atta array

## Abstract

This paper proposes a 2-D fully polarized Van Atta array, which consists of four tri-polarized antenna elements. The tri-polarized antenna element comprises a monopole antenna and a low-profile microstrip antenna that widens the beam by folding four electric walls. This configuration enables the Van Atta arrays to receive and transmit arbitrarily polarized incident waves over a wider range. The measurement results indicate that the proposed Van Atta array exhibits a −5 dB radar cross-section (RCS) greater than 95° when TE-polarized waves are incident and greater than 134° when TM-polarized waves are incident, significantly surpassing the 2-D dual-polarized array.

## 1. Introduction

A retrodirective array (RDA) is an adaptive antenna array that can adjust the array beam pointing towards the incoming wave direction based on received incident wave information through a phase-conjugated circuit without requiring complex prior knowledge [1,2,3], which has been widely used in wireless sensing technology [4], automobile collision avoidance systems [5], and radio frequency identification systems [6]. According to the implementation form of phase conjugation, retrodirectivity can be achieved by using corner reflectors, Van Atta arrays, phase-conjugated arrays, phase-locked loop-based retrodirective arrays, digital conjugate circuit-based retrodirective arrays, and other types. Among these, the Van Atta array is of particular interest due to its simple structure and ability to achieve a relatively larger radar cross-section (RCS) across a wide range of incidence angles [7]. In the Van Atta array, each pair of transmitting and receiving antennas is symmetrically distributed along the geometric center of the array and connected by transmission lines of equal electrical length. When the receiving antenna detects a signal, it transmits the signal through the line to the transmitting antenna, resulting in an inverse phase between the received and transmitted signals. Consequently, the transmit antenna beam points toward the direction of the incoming wave, achieving retrodirectivity. Typically, each antenna element receives the incident wave and also transmits the retrodirective wave.

The retrodirective range is a key performance indicator for retrodirective arrays. For the Van Atta array, this range is primarily linked to the beamwidth of the array elements, enabling a wider retrodirective angle through the use of wide-beam antenna elements. Many excellent performance wide-beam antennas have been designed by scholars in recent years, In [8], a ±45° dual-polarized dipole element antenna was designed by placing two orthogonally coupled feed baluns on a dual-polarized dipole, thereby exciting the two pairs of orthogonal arms of the dipole, resulting in an antenna with a beamwidth in excess of 100°. In [9], a series of conductive posts and ground slots are used to increase the 3 dB beamwidth of a microstrip antenna to 130°. In [10], the authors increased the E-plane/H-plane beamwidth of the microstrip antenna to 180°/120° by adding four mushroom-shaped monopole patches around the microstrip antenna. However, most of them broaden the antenna beam by using the method of adding the three-dimensional structure, which limits the applicability of these antennas in Van Atta arrays due to their high profile. The performance of Van Atta arrays is not only affected by the beamwidth of the array cells but is also related to the polarization mode of the array cells. Traditional Van Atta arrays have been designed and applied using single-linear polarization antennas such as dipole antennas [11], patch antennas [12,13], and slot antennas [14,15], which will lead to polarization mismatch when receiving incident electromagnetic waves perpendicular to their polarization direction, resulting in reduced RDA efficiency. Although the polarization mismatch issue caused by single-linear polarization antennas has been overcome by using dual-polarized antennas in [16,17,18,19,20], dual-polarized antennas remain incapable of fully receiving an incident wave of arbitrary polarization. Dual-polarized microstrip antenna arrays are unable to function correctly when the incident wave is in the end-fire direction. This is due to the fact that microstrip antennas are only capable of receiving and retrodirecting signals in the broadside direction. In order to address the issue of polarization mismatch, a Van Atta array based on tri-polarized antenna elements is proposed in [21], which greatly extends the range of retrodirective arrays, enabling them to retrodirect incident waves of arbitrary polarization. However, their work is limited to the one-dimensional array, resulting in retrodirectivity that can be achieved in one direction, which also limits the application scenarios of the Van Atta array.

To address the aforementioned issues, this paper presents a planar Van Atta array based on wide-beam tri-polarized antenna elements. Although many high-performance tri-polarized antennas have been proposed in previous works [22,23,24], most of them are large in size and do not exhibit wide-beam characteristics. If these antennas were applied to a planar Van Atta array, they would undoubtedly limit the retroreflective range of the array. The tri-polarized antenna elements designed in this paper combine both wide-beam and compact-size features, significantly enhancing the retroreflective range of the Van Atta array and addressing the polarization mismatch problem.

In this paper, a wide-beam orthogonal dual-polarized microstrip antenna element was developed using the superposition principle, leading to the design of a wide-beam triple-polarized antenna element. Building on this, a 2-D fully polarized Van Atta array comprising four wide-beam tri-polarized antenna elements is proposed, capable of receiving plane waves of arbitrary polarization and retrodirecting them over a wide range. Measurements of the monostatic RCS indicate that the 2-D Van Atta array utilizing wide-beam tri-polarized elements demonstrates superior retrodirective range characteristics compared to the dual-polarized version for both transverse-electric (TE) and transverse-magnetic (TM) polarized incident waves.

## 2. Theory and Design

### 2.1. Design and Measurement of Wide-Beam Tri-Polarized Antenna

**Figure 1:**
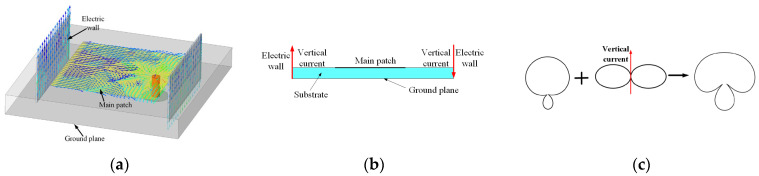
Schematic diagram of the antenna loaded with electric walls: (**a**) current relationship between vertical electric walls and main patch; (**b**) electric wall loading method; (**c**) principle of beam spreading.

In this paper, the beamwidth of a microstrip antenna is expanded by incorporating a vertical electric wall. As illustrated in Figure 1, the induced currents are generated on the wall due to coupling when the vertical electric walls are close to the main patch. The vertical current coupled with the main patch on the vertical electric wall generates radiation in the horizontal direction, which combines with the radiation pattern of the main patch, effectively broadening the beamwidth of the antenna.

**Figure 2:**
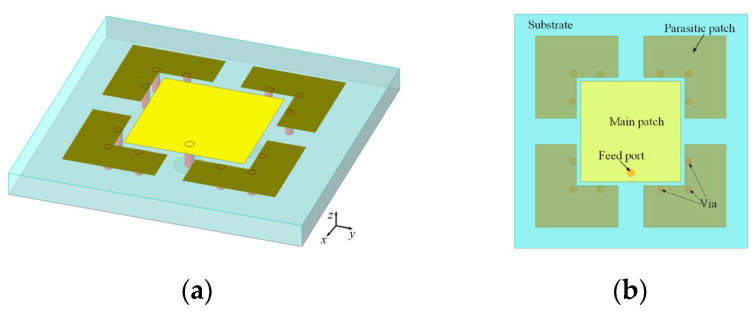
Antenna loaded with parasitic patches: (**a**) 3D view; (**b**) top view.

To minimize the profile of the antenna, this paper employs the technique of folding the electric wall. As shown in Figure 2, four parasitic patches are introduced on the dielectric surface, and then these parasitic patches and the ground plane are connected using 12 metal vias, resulting in a dielectric-integrable wide-beam antenna design.

**Figure 3:**
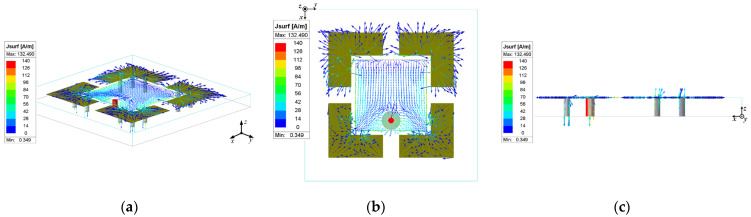
Current flow on the surface of the antenna loaded with folded electric walls: (**a**) 3D view; (**b**) top view; (**c**) side view.

As shown in Figure 3, the main patch couples an induced current on the parasitic patch by loading the folded electric wall. This induced current flows to the ground through the metal vias (or from the ground to the parasitic patches), thereby generating the vertical currents on the metal vias. The metal vias produce a radiation pattern as shown in Figure 1c, which overlaps with the radiation pattern of the main patch, thus broadening the antenna beam.

**Figure 4:**
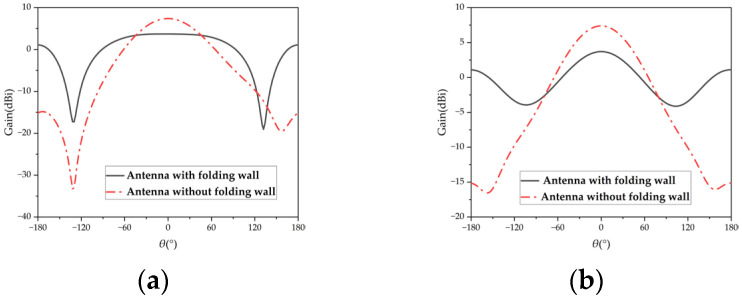
Comparison of simulated results of antenna pattern with or without a folding wall: (**a**) E-plane pattern; (**b**) H-plane pattern.

As depicted in Figure 4, the introduction of the folding wall results in an E-plane beamwidth of approximately 174° and an H-plane beamwidth of 95° for the antenna. In contrast, without the folding wall, the antenna exhibits an E-plane beamwidth of only 77° and an H-plane beamwidth of 80°. These findings demonstrate that the inclusion of the folding wall significantly broadens the beamwidth of the microstrip antenna, particularly in the E-plane.

Figure 4 illustrates that after implementing the folded electric wall, the microstrip antenna significantly widens its E-plane beam, while the H-plane beamwidth remains largely unchanged. This difference arises because the E-plane and H-plane are perpendicular. The same electric wall loading method was applied to both planes, resulting in reverse currents on the H-plane’s electric walls that cancel each other’s radiation patterns, preventing H-plane widening. To resolve this, staggering the vertical electric wall in the H-plane can ensure that the radiation patterns from the vertical currents do not fully cancel out.

**Figure 5:**
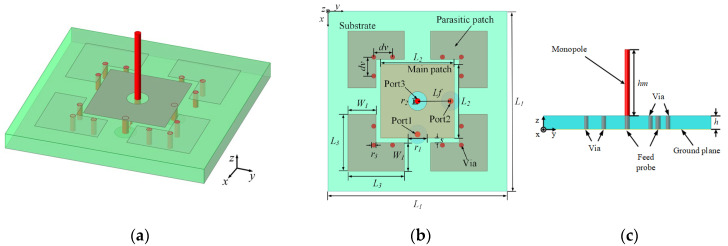
Geometry of tri-polarized antenna: (**a**) 3D view; (**b**) top view; (**c**) side view (L1 = 19 mm, L2 = 7.8 mm, L3 = 6 mm, r1 = 2 mm, r2 = 0.51 mm, r3 = 0.5 mm, dv = 2 mm, S = 0.5 mm, W1 = 2.5 mm, h = 1.5 mm, hm = 7.5 mm, Lf = 3.5 mm).

As shown in Figure 5, the tri-polarized antenna is implemented by combining a dual-polarized microstrip antenna with a monopole antenna in this paper. The square microstrip patch antenna excites the TM10 mode and TM01 mode, both of which have a zero electric field at the center of the patch. Therefore, a monopole antenna can be placed at the center of the patch by creating a properly sized opening, without significantly impacting the performance of the microstrip antenna. An F4B substrate (ε_r_ = 2.2 and tanδ = 0.001) with a thickness of 1.5 mm is utilized.

**Figure 6:**
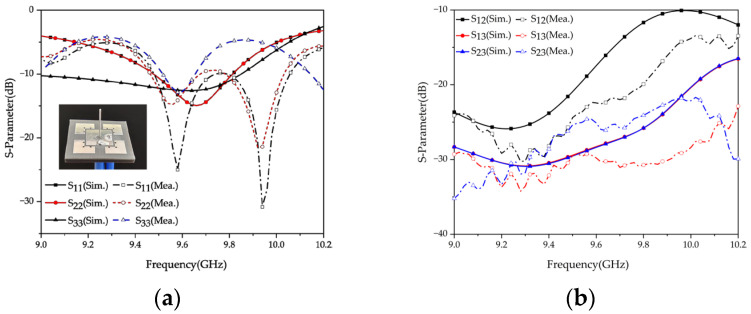
Simulated and measured S-parameters of tri-polarized antennas: (**a**) reflection coefficients; (**b**) isolations.

The simulated and measured results of the reflection coefficient for the tri-polarized antenna are presented in Figure 6a. The reflection coefficients of the antenna are below −10 dB within the frequency range of 9.52–9.68 GHz. Additionally, Figure 6b illustrates the simulated and measured isolation between each port. The isolation between ports exceeds 15 dB within the operating bandwidth of the antenna.

**Figure 7:**
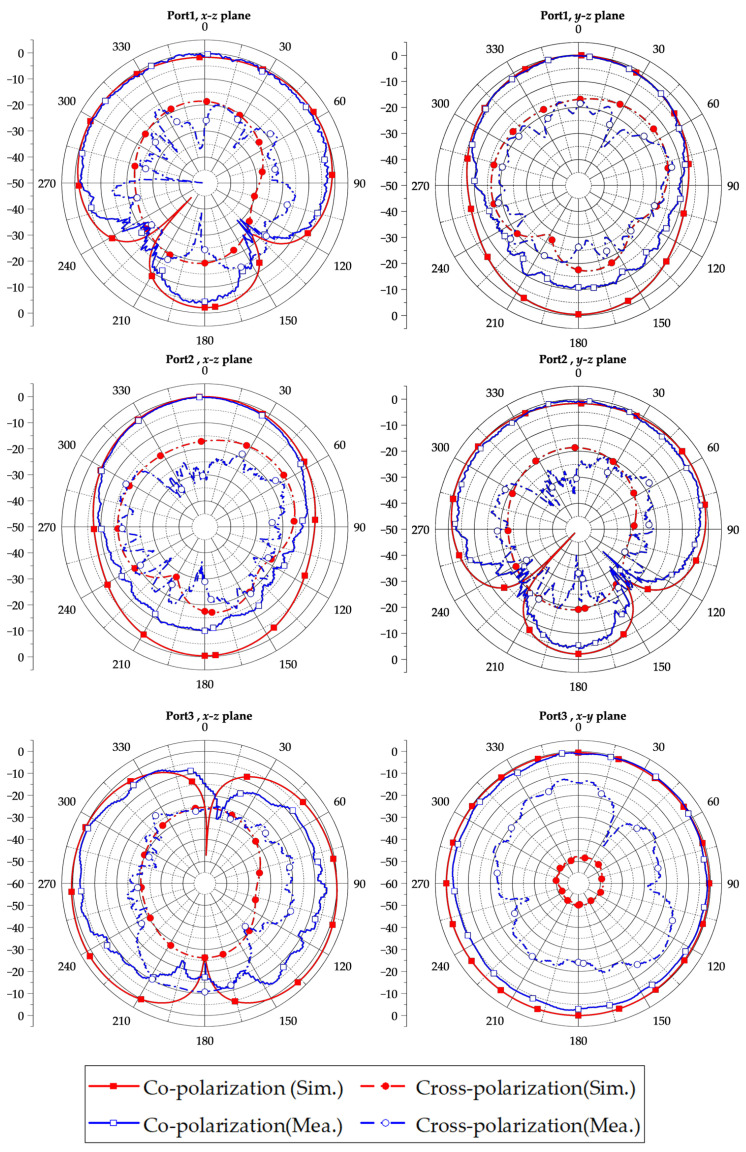
Simulated and measured radiation patterns at 9.6 GHz of the proposed antenna.

Figure 7 illustrates the simulated and measured radiation patterns of the tri-polarized antenna. When port 1 is excited, the simulated/measured beamwidth in the E-plane (xoz plane) is 205°/181.64°, and the simulated/measured beamwidth is 96°/94.72° in the H-plane (yoz plane). When port 2 is excited, the simulated/measured beamwidth in the H-plane (xoz plane) is 96°/90.58°, and in the E-plane (yoz plane), it is 205°/180.19°. It is worth noting that the measured beamwidth of the E-plane directional pattern is 24° smaller than the simulated result, which can be attributed to factors such as soldering and the influence of the measurement environment. When port 3 is excited, the antenna produces a radiation pattern resembling that of a monopole with omnidirectionality in the xoy plane.

Table 1 presents a comparison of the wide-beam tri-polarization antenna designed in this study with various antennas from the references. As shown in Table 1, existing tri-polarization antennas typically utilize a narrow-beam design. In contrast, this research is the first to apply wide-beam antenna technology to tri-polarization antennas, achieving widened beamwidth while maintaining a compact antenna size compared to traditional single-polarized or dual-polarized wide-beam antennas.

### 2.2. Design and Measurement of the Antenna Array

**Figure 8:**
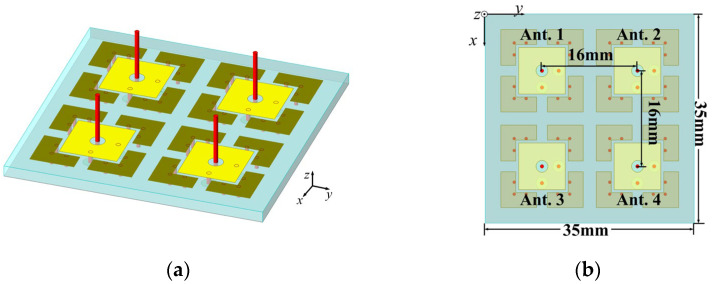
Prototype of the antenna array: (**a**) 3D view; (**b**) top view.

For the Van Atta array, the retrodirective range of the array primarily depends on the beamwidth of the array elements. The number of elements in the array does not significantly affect the retrodirective range. Therefore, this paper employs the simplest 2 × 2 array configuration. The structure of the 2-D antenna array proposed in this paper is shown in Figure 8; the spacing between array elements is set to 16 mm (0.512 *λ*_0_) to ensure proper performance. The overall volume of the 2-D antenna array is 1.12 × 1.12 × 0.288 $\lambda_{0}^{3}$.

**Figure 9:**
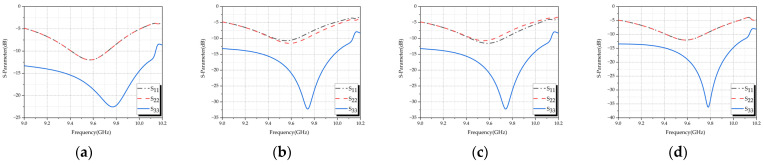
Reflection coefficients of antenna array elements: (**a**) Ant. 1; (**b**) Ant. 2; (**c**) Ant. 3; (**d**) Ant. 4.

To ensure that there is no frequency offset caused by mutual interference between the elements during the formation of the array, the reflection coefficients of each element in the antenna array were simulated individually. The results of the simulations are shown in Figure 9. It can be observed that the operating bandwidth of each port of the elements in the designed array is similar to that of the independent elements, indicating that the operating frequency of the antenna array is consistent with that of the antenna elements.

### 2.3. Implementation and Performance of Van Atta Array

**Figure 10:**
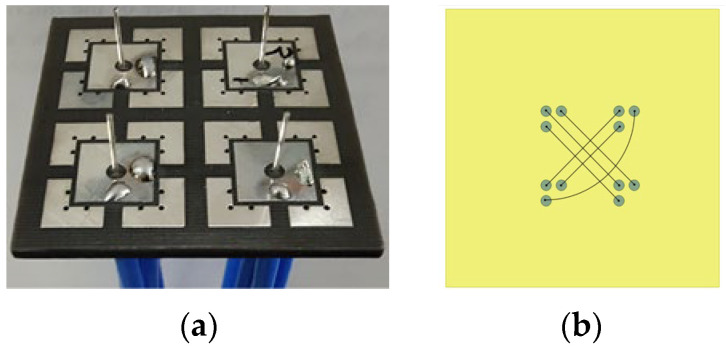
Van Atta array: (**a**) prototype of the Van Atta array; (**b**) connections for fully polarized planar Van Atta array.

The model of the fully polarized Van Atta array designed in this paper is shown in Figure 10, and Figure 10a shows the array prototype. The transmission line connections of the planar Van Atta array are depicted in Figure 10b. Each antenna element is connected to its corresponding element using an equal electrical length transmission line. In the case of the tri-polarized antenna element, the same polarization ports of the corresponding antennas are connected accordingly through the equal electrical length transmission line, enabling retrodirectivity to be achieved. To simplify the design, this paper employs equal-length coaxial cables to connect each corresponding port within the array to ensure that each corresponding port satisfies the phase requirement relationships.

**Figure 11:**
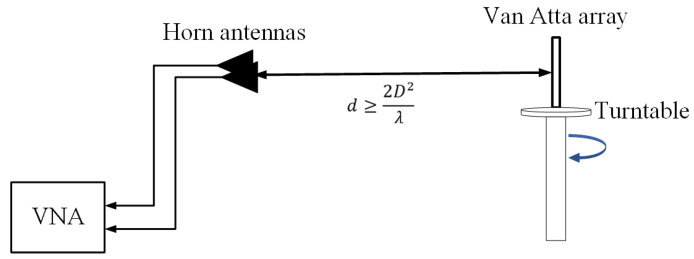
Schematic of the measurement system for the monostatic RCS.

Figure 11 shows the measurement system schematic for the monostatic RCS, comprising a turntable, two horn antennas, and a vector network analyzer (VNA). One horn acts as the transmitter and the other as the receiver, with both connected to the VNA. Positioned side by side in a horizontal plane, the antennas face the Van Atta array, which must be at least $\frac{{2D}^{2}}{\lambda }$ away to ensure plane wave incidence, where D is the array’s maximum size and *λ* is the wavelength. The array is mounted vertically on the turntable, aligned with the antenna centers. During measurements, the incident wave’s polarization is adjusted by modifying the horn antenna’s orientation, allowing for tests of the Van Atta array’s monostatic RCS at different polarization angles. A TM-polarized wave is defined as parallel to the turntable, while a TE wave is perpendicular to it.

**Figure 12:**
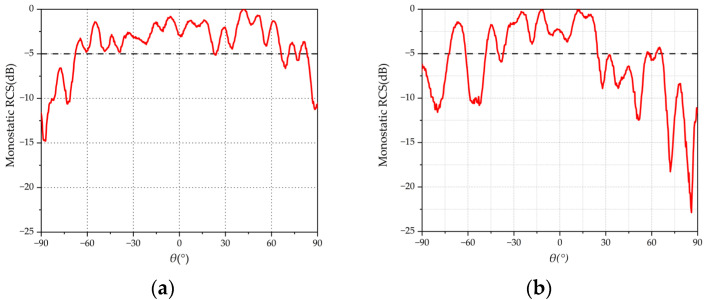
Measured monostatic RCS with TM incident wave: (**a**) tri-polarized antenna; (**b**) dual-polarized antenna.

**Figure 13:**
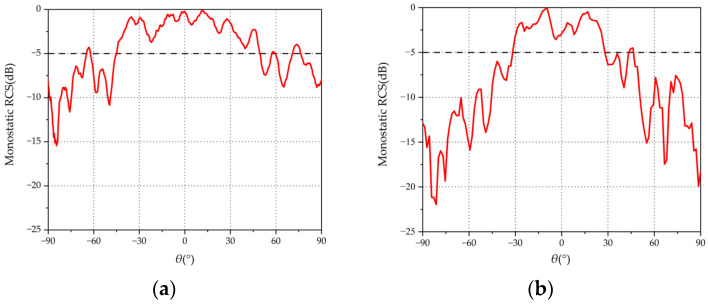
Measured monostatic RCS with TE incident wave: (**a**) tri-polarized antenna; (**b**) dual-polarized antenna.

The measured monostatic RCSs of the tri-polarized and dual-polarized arrays for TM- and TE-polarized wave incidences at 9.6 GHz are given in Figure 12 and Figure 13. From Figure 12, it can be observed that, for the tri-polarized Van Atta array with a TM incident wave, the range of measured monostatic RCS greater than −5 dB is 134.41°, and the dual-polarized array is only 73.11°. As shown in Figure 13, For the TE incident, the measured −5 dB beamwidth for the tri-polarized Van Atta array is 95.15°, while the dual-polarized array achieves a beamwidth of only 60.1°. These results indicate that the 2-D Van Atta array based on wide-beam tri-polarized antenna elements can achieve a wide retrodirective range for incident waves incident along any polarization, and the tri-polarized Van Atta array outperforms the dual-polarized Van Atta array by a significant margin.

## 3. Discussion

This paper presents the design of a wide-beam orthogonal dual-polarized microstrip antenna based on the principle of pattern superposition. The profile of the designed wide-beam antenna is reduced using the method of folded electric walls. A tri-polarized antenna with wide-beam characteristics is developed by combining the wide-beam orthogonal dual-polarized microstrip antenna with a monopole antenna. Building on the aforementioned antenna elements, this study proposes and designs a planar Van Atta array based on wide-beam tri-polarized antenna elements for the first time and measures its monostatic RCS under different plane wave incidences. The measurement results indicate that the Van Atta array, based on wide-beam tri-polarized antenna elements, can effectively accept and reflect incident waves of any polarization over a wide range, addressing the polarization mismatch issue caused by the use of single-polarized or dual-polarized elements in existing Van Atta arrays.

Based on Table 2, it can be observed that the proposed planar Van Atta array, utilizing wide-beam tri-polarized antenna elements, effectively enhances the retrodirective performance of the Van Atta array compared to single- or dual-polarized Van Atta arrays, while also featuring a more compact structure.

The measurement results of the monostatic RCS indicate that the Van Atta array using tri-polarized antenna elements exhibits a superior retrodirective range compared to those using dual-polarized antenna elements. This advantage arises because the maximum radiation field of the dual-polarized microstrip antenna is primarily concentrated around θ = 0°. As θ increases to ±90°, the microstrip antenna is nearly unable to receive incident signals, significantly limiting the retrodirective range of the Van Atta array. In contrast, the monopole antenna has maximum gain in the θ = ±90° directions, allowing it to function effectively when the incident angle of the plane wave increases to ±90°. Therefore, the designed wide-beam tri-polarized antenna element not only possesses a broad beam in the upper half-plane but can also receive and retrodirect plane waves incident at θ = ±90°, thereby increasing the retrodirective range of the planar Van Atta array. The proposed planar Van Atta array addresses the polarization mismatch issue faced by traditional Van Atta arrays, thereby further expanding the application scenarios for Van Atta arrays.

To enhance the effectiveness and broaden the applicability of this research, further investigations will be conducted in the following areas:

(1) Improvements will be made to the orthogonal dual-polarized microstrip antenna to achieve broader beamwidths in both the E-plane and H-plane.
(2) A study will be conducted on low-profile, miniaturized planar monopole antennas, applying them to wide-beam tri-polarized antennas to enhance structural stability and reduce profile height.
(3) The transmission line of the planar Van Atta array will be optimized to lower the overall profile of the array.

Furthermore, with the advancement of modern communication technology, there are increasing demands for antenna bandwidth. Therefore, designing a Van Atta array based on wide-bandwidth beam tri-polarized microstrip antenna elements will also be a key focus of future research.

## 4. Conclusions

A 2-D Van Atta array based on wide-beam tri-polarized antennas is proposed in this paper. By leveraging three orthogonal polarization modes that complement each other, the tri-polarized Van Atta array achieves excellent retrodirective performance for incident waves across a wide range of angles. The measured monostatic RCS results demonstrate a −5 dB retrodirective range of 134.41° with TE incidence and a −5 dB retrodirective range of 95.15° with TM incidence. The proposed 2-D Van Atta array holds promise for applications in target identification systems, radar systems, wireless communication systems, and more.

## Figures and Tables

**Table 1:** Comparison of discussed antennas and this study.

Type	Ref.	Polarization	Frequency (GHz)	Bandwidth (GHz)	Beamwidth	Isolation (dB)	Size (L × W × h)
Wide-beam antenna	[8]	double	24–33	41.6%	>100°	>25	1.8 × 1.8 × 0.14 $\lambda_{0}^{3}$
	[9]	single	2.16–2.24	3.6%	130°	-	π × 0.19 × 0.19 × 0.26 $\lambda_{0}^{3}$
	[10]	double	2.36–2.71	13.8%	E-plane > 180°	>43	0.83 × 0.83 × 0.096 $\lambda_{0}^{3}$
					H-plane > 120°		
Tri-polarized antenna	[21]	triple	5.78–5.82	2.5%	narrow	>15	0.476 × 0.476 × 0.039 $\lambda_{0}^{3}$
	[22]	triple	2.3–2.5	10.4%	narrow	>22	0.664 × 0.664 × 0.128 $\lambda_{0}^{3}$
	[23]	triple	2.04–2.26	10.47%	narrow	>13	0.77 × 0.77 × 0.07 $\lambda_{0}^{3}$
	[24]	triple	14.42–17.44	19%	narrow	>15	0.65 × 0.65 × 0.349 $\lambda_{0}^{3}$
This work	-	triple	9.52–9.68	1.6%	E-plane > 181°	>14	0.63 × 0.63 × 0.288 $\lambda_{0}^{3}$
					H-plane > 90°		

**Table 2:** Comparison of discussed Van Atta arrays and this study.

Ref.	Type of Array	Polarization	Frequency (GHz)	Unit Number	Monostatic RCS Beamwidth (°)	Size (L × W × h)
[12]	planar	single	5.75 and 11.4	25	87° (−4 dB@5.75 GHz)	2.98 × 2.98 × 0.068 $\lambda_{0}^{3}$
					75° (−4.5 dB@11.4 GHz)	
[14]	planar	single	5.8	32	50° (−3 dB)	3.52 × 1.76 × 1.04 $\lambda_{0}^{3}$
[20]	planar	double	2	4	80° (−3 dB)	1.69 × 1.86 × 0.01 $\lambda_{0}^{3}$
[21]	linear	triple	5.6	4	132° (−3 dB)	1.93 × 0.56 × 0.059 $\lambda_{0}^{3}$
This work	planar	triple	9.6	4	134.41° (−5 dB)	1.12 × 1.12 × 0.288 $\lambda_{0}^{3}$
					108.57° (−3 dB)	

## Data Availability

The datasets presented in this article are not readily available because the data are part of an ongoing study. Requests to access the datasets should be directed to jcpan0719@163.com.
